# Image recovery from unknown network mechanisms for DNA sequencing-based microscopy†

**DOI:** 10.1039/d2nr05435c

**Published:** 2023-04-14

**Authors:** David Fernandez Bonet, Ian T. Hoffecker

**Affiliations:** a Science for Life Laboratory, Department of Gene Technology, KTH Royal Institute of Technology Tomtebodavägen 23a 171 65 Solna Sweden ithof@kth.se +46725024619

## Abstract

Imaging-by-sequencing methods are an emerging alternative to conventional optical micro- or nanoscale imaging. In these methods, molecular networks form through proximity-dependent association between DNA molecules carrying random sequence identifiers. DNA strands record pairwise associations such that network structure may be recovered by sequencing which, in turn, reveals the underlying spatial relationships between molecules comprising the network. Determining the computational reconstruction strategy that makes the best use of the information (in terms of spatial localization accuracy, robustness to noise, and scalability) in these networks is an open problem. We present a graph-based technique for reconstructing a diversity of molecular network classes in 2 and 3 dimensions without prior knowledge of their fundamental generation mechanisms. The model achieves robustness by obtaining an unsupervised sampling of local and global network structure using random walks, making use of minimal prior assumptions. Images are recovered from networks in two stages of dimensionality reduction first with a structural discovery step followed by a manifold learning step. By breaking the process into stages, computational complexity could be reduced leading to fast and accurate performance. Our method represents a means by which diverse molecular network generation scenarios can be unified with a common reconstruction framework.

## Abstract 2

Optimal image reconstruction is an open problem in the field of imaging-by-sequencing. We implement an algorithm that is scalable and robust to different network types by using structural discovery.

## Graphical abstract

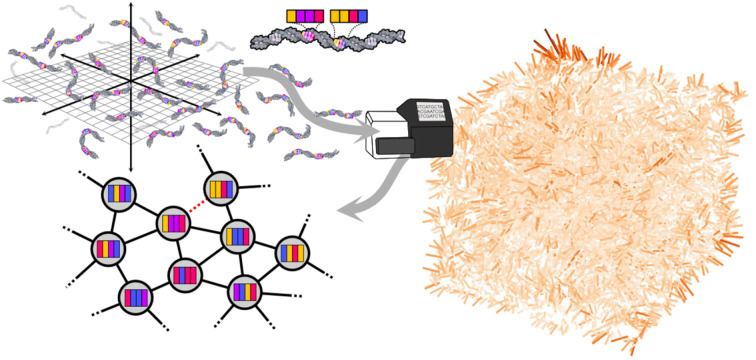

Imaging-by-sequencing methods^1–9^ arose recently as a potential alternative molecular imaging strategy based entirely on DNA sequencing information in contrast to optical or optics-coupled techniques like spatial omics,^10–17^ single molecule localization microscopy,^18–21^ or fluorescence imaging more broadly. Individual nanoscale molecular associations in imaging-by-sequencing lead to unique sequence-based records that denote local proximity between molecules. This notion of proximity-dependent association is extended to form large interconnected networks that encompass a global space (Fig. 1). By relating network structure to spatial location, whole images can be obtained, as demonstrated experimentally in an approach where images of cultured cells were reconstructed on the basis of network-representing sequencing data alone without the use of classical optical microscopy.^5^ We can formally represent this spatial information as a graph, where strands are nodes and strand-to-strand associations are edges. An open computational problem is that of optimal image reconstruction, *i.e.* the task of how to quickly and accurately map strand-to-strand associations into a spatial representation reflecting the underlying micro- or nanoscale molecular distribution.

**Fig. 1:**
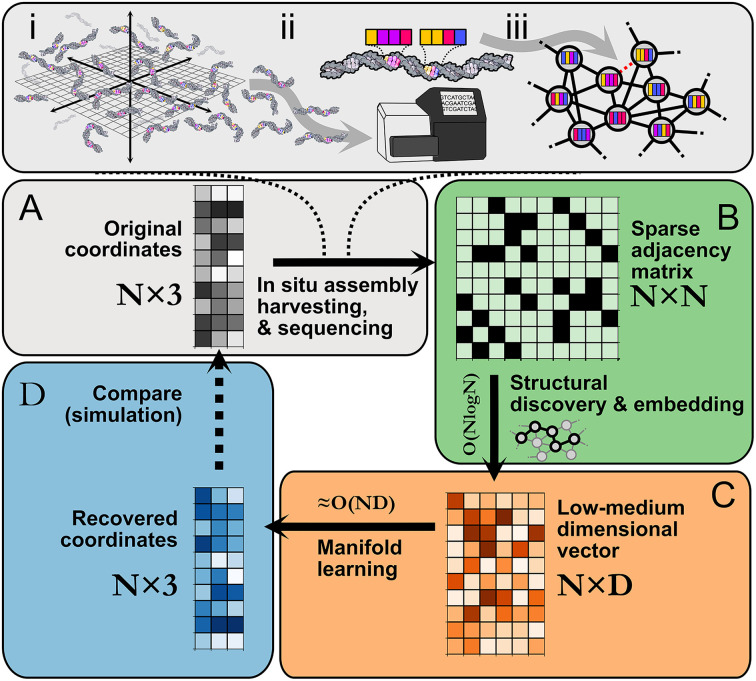
Imaging-by-sequencing by structural embedding: (A) (i) *in situ* network formation, (ii) harvesting, sequencing, and cataloging of network edges, (iii) network re-assembly. (B) Beginning from an *N* × *N* pairwise binary adjacency matrix, an initial structural discovery step uses random walk sampling to compress the data into an *N* × *D* matrix. (C) The remaining dimensionality reduction is carried out with manifold learning. (D) The recovered *N* × 3 or *N* × 2 set of coordinates is an approximation of the original (unknown) set of relative molecular positions.

A number of different imaging-by-sequencing strategies have been proposed along with different corresponding network generating rules. For example: networks where nodes are connected to most other nodes,^5^ locally connected Voronoi meshes,^6^ locally connected neighbor networks,^3,4,7,8^ “GPS” networks,^9^ or boundary-sharing cell networks.^1,2^ Strategies moreover fall into distance-weighted^5,8,9^ or binary unweighted categories^3,4,6^ (ESI section 1.A.2†). These design differences at the fundamental structure level seem to suggest that reconstruction strategies must be tailored to the network generation strategy. In practice, one lacks complete knowledge of the microscopic processes driving network formation which are likely more complex than their design. Existing reconstruction strategies thus rely on models of network formation that only partially reflect reality. Scaling difficulties also arise whereby reconstruction becomes either prohibitively slow or memory-intensive with large matrices, *e.g.* in eigendecomposition of a data matrix. Finally, most strategies optimize for objectives that only coincide approximately with spatial localization (*e.g.* spring relaxation approaches) without being explicitly tied to it or rely on a rigid definition of neighborhood. This results in an insensitivity to different connectivity patterns that could arise either by design, through noise, or imprecise assumptions about the network generation process. An alternative approach is to learn, in an unsupervised format, those representations of each node in a spatial network that optimize for neighborhood similarity prediction. In this study, we achieve robust, scalable spatial reconstruction from a diversity of network formation patterns despite minimal prior knowledge of the underlying generating rules by performing Spatio-topological recovery by network discovery (STRND).

Following *in situ* self assembly, harvesting, and sequencing of a spatial DNA network (Fig. 1A), STRND is a pipeline that begins from a pairwise adjacency matrix of the form1
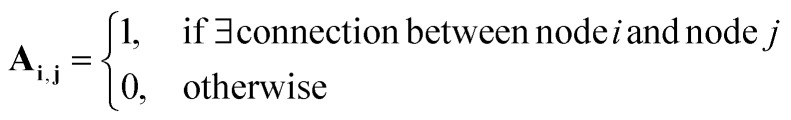


STRND reduces the dimensionality of the initial *N* × *N* matrix in two stages. First, we perform a network structure discovery step based on graph representation learning.^22^ In particular, random walks are used to sample the local and global structural characteristics in the neighborhood of each node in the graph to produce a node embedding (Node2Vec,^23^Fig. 1B). The output yields *N* multi-dimensional vectors of dimension *D* (feature vectors) such that typically *D* ≪ *N*. Second, feature vectors are fed into a subsequent dimensionality reduction stage (Fig. 1C) that uses manifold learning to embed vectors into either 2 or 3 dimensions to restore the initial positions of the DNA network.

These reconstructed points (Fig. 1D) approximate the original image within some accuracy. All reconstructions are obtained using hyperparameter values which are found to be a compromise between reconstruction accuracy and low computational complexity (Table S1†).

Reducing dimensionality in stages improves computational complexity, as manifold learning can be computationally demanding. Compressing the adjacency matrix through node embedding results in a lower analytical computational complexity for manifold learning, which becomes near-linear ≈*O*(*N*) since *D* ≪ *N* as will generally be the case for large networks. Furthermore, the structural discovery step has an upper bound analytical time complexity *O*(*N* log *N*), a major improvement compared to directly applying manifold learning with complexity *O*(*ND*), where *D* = *N*.

Redistributing tasks is a common strategy to reduce overall complexity. For example, an aesthetic graph drawing method^24^ leverages shortest-path distances from key nodes for structural discovery, while subsequent reduction is carried out with Principal Component Analysis (PCA).^25^ Landmark Isomap^26,27^ also reduces complexity by establishing a selected number of landmarks and computing the shortest-path distance from every node to every landmark. In contrast, STRND uses a random walk node embedding to achieve a spatial representation of each node with low space complexity per node. Because we desire strict preservation of all geometric relationships, we use Uniform Manifold Approximation and Projection (UMAP) over techniques such as PCA for its superior preservation of local and global geometry.^28^ Reconstruction accuracy and empirical computational complexity are compared between approaches in ESI section 3.†

Using structural discovery followed by manifold learning, we reconstructed 2 and 3-dimensional simulated point distributions (Fig. 2A). Initial molecule positions were randomly distributed over the space of a square or cube of characteristic length *L* = 1 with no prior assignment of molecule identity to position so as to model DNA dispersion in an imaging-by-sequencing experiment.

**Fig. 2:**
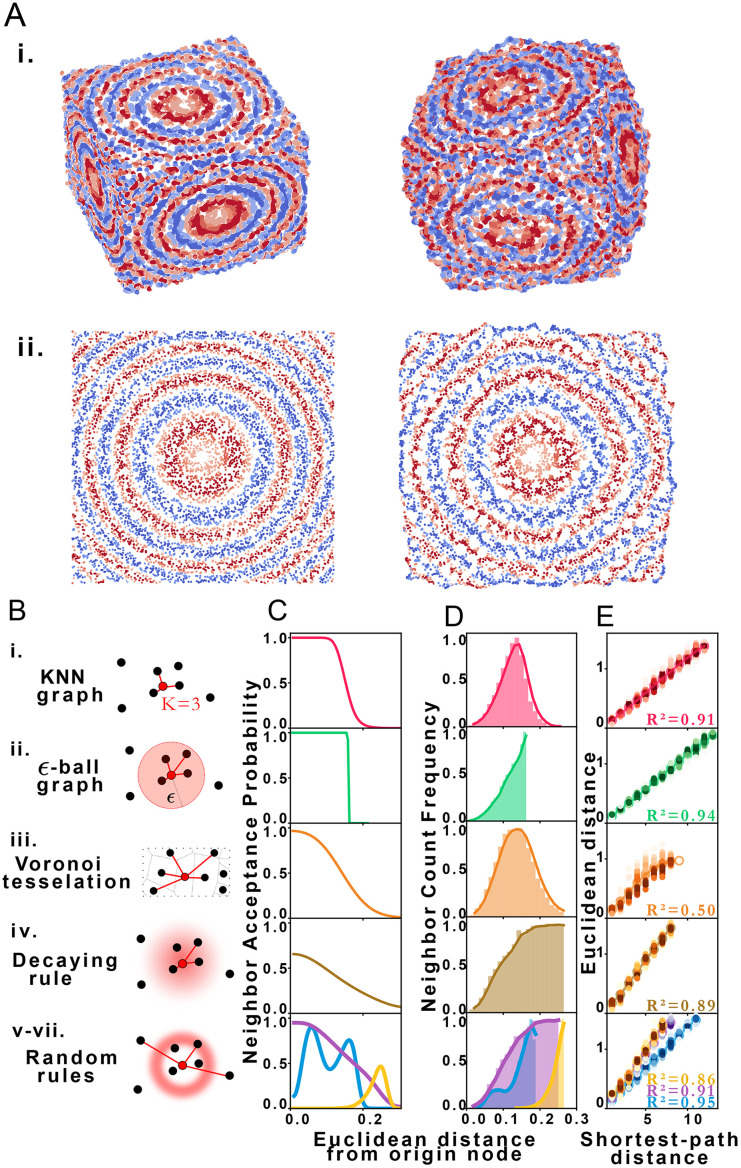
STRND reconstruction. (A) Original (left) set of points and resulting reconstructed (right) points in i. 3 dimensions and ii. 2 dimensions. (B) Types of proximity graphs (i–vii) and corresponding edge drawing rules with probability clouds for stochastic rules. (C) Probability that nodes *i* and *j* are neighbors (share an edge) given that their Euclidean distance is *d*_*ij*_ for each type of proximity graph (i–vii) respectively. (D) Neighbor count frequency with distance. (E) Correlation between Euclidean distance and shortest-path distance for each network type (i–vii) respectively.

We explored STRND's robustness to variation in network structure by choosing multiple rule sets. Each rule represents a different model of physical association (Fig. 2B), *i.e.* different proximity to edge mappings. Proximity graph definitions^29^ are summarized in Table 1. We explored 3 deterministic (KNN-graph, *ε*-ball graph, Voronoi tessellation) and 4 stochastic proximity graphs (based on probabilistic rules). For completeness, we examined a KNN distance-weighted graph in contrast to the unweighted graphs represented by eqn (1), whereby edges are weighted by some function of separation distance (in this case the inverse distance).

**Table 1:** Proximity graph rules

Proximity graph	Rule: connect origin node to candidate…
i. KNN graph	If among the k closest neighbors
ii. *ε*-Ball graph	If within distance *ε* to origin node
iii. Voronoi tessellation	If Voronoi cell shares border with origin cell
iv. Decaying rule	According a distance-decaying probability
v–vii. Random rules	According to arbitrary probability distribution

Network generation rules exhibit characteristic neighbor acceptance probability distributions as a function of distance between neighbor and origin node (Fig. 2C). For an arbitrary set of randomly distributed points, different rules produce distinct neighbor frequency distributions, *i.e.*, (normalized) number of neighbors encountered as a function of distance from a given node (Fig. 2D). We observed that all network rules gave rise to monotonic relationships between the average Euclidean distance and graph shortest-path distance (Fig. 2E). This observation suggests a basis for geometry preservation between Euclidean and graph space, *i.e.*, there is an expected Euclidean distance corresponding to each shortest-path distance in a given reconstructed network. A geometric relationship between a set of points represented as a set of shortest-path distances in graph space may thus be expected to have a corresponding (though probabilistic) relative geometric relationship in Euclidean space due to this mapping.

Ground truth access *via* simulation enables us to compare original and reconstructed points to assess accuracy. We quantify accuracy according to three standards: a local, a global, and a mean distortion quality metric. The local quality metric (KNN, ESI section 2.D.2†) examines the difference between original and reconstructed neighborhoods of every point. We use *K* = 15 neighbors inspired by the average number of neighbors of Voronoi tesselations in 3D. Overall, the KNN metric is an indicator of fine structure preservation. Conversely, the global quality metric (CPD, ESI section 2.D.3†) examines the pairwise distance Pearson correlation between original and reconstructed points and is an indicator of coarse structure. Lastly, the mean distortion is obtained *via* affine transformation (ESI section 2.D.4†) on the reconstructed points. We define distortion as the displacement between original and reconstructed points, a lower value indicative of better reconstruction, with the mean obtained by averaging the distortion of all points.

Fig. 3A shows a visualization of distortion following reconstruction of 10 000 points for 2D and 3D cases. While central points show below-average distortions, border points exhibit higher distortions, which we attribute to anisotropic topology near the boundaries in contrast to the isotropic core of the point cloud. Reconstruction accuracy dependence is measured *via* three parameters: dimension, system size and proximity graph type (Fig. 3B). Greater system sizes correspond to points being more densely packed in space, although the average number of accepted neighbors is similar. Importantly, accuracy in all categories varies minimally by proximity graph type (weighted or unweighted) as shown in Table S7.† Stochastic proximity rules exhibit stable quality trends in line with the other graph types. Local reconstruction quality according to the KNN metric (Fig. 3Bi and iv) 2D reconstruction was robust to proximity graph type, with a maximum variation of 1.5% and 0.7% in the case of 3D. The global quality metric (Fig. 3Bii and v) showed that pairwise distances between original and reconstructed points were linearly correlated, with a correlation coefficient near 1. This indicates that relative distances were preserved during reconstruction. Global quality in the 2D case exhibits the largest variation to proximity graph type, with a maximum of ≈6%, whereas the maximum variation in 3D was an order of magnitude lower at 0.6%. Distortion also does not vary much with proximity graph type (Fig. 3Biii and vi). However, in agreement with the other metrics, distortion worsens with increasing points. An exception to this tendency happens when the system size is small enough for the 3D case (*N* = 1000), with an improved distortion for higher sizes. Random walk length and embedding dimension (*i.e.* size of the output feature vector) needed to be increased with greater system size to maintain accuracy. We attribute this to increased demands for representing spatial information in larger systems, *e.g.* encoding not only immediate neighborhoods but also communities of nodes (ESI section 2.C†). Overall we note that the pipeline works without user supplied knowledge of the network, as this is managed automatically in the unsupervised structural discovery stage. This would be advantageous in an experimental setup with imperfect knowledge of the molecular processes leading to proximity associations.

**Fig. 3:**
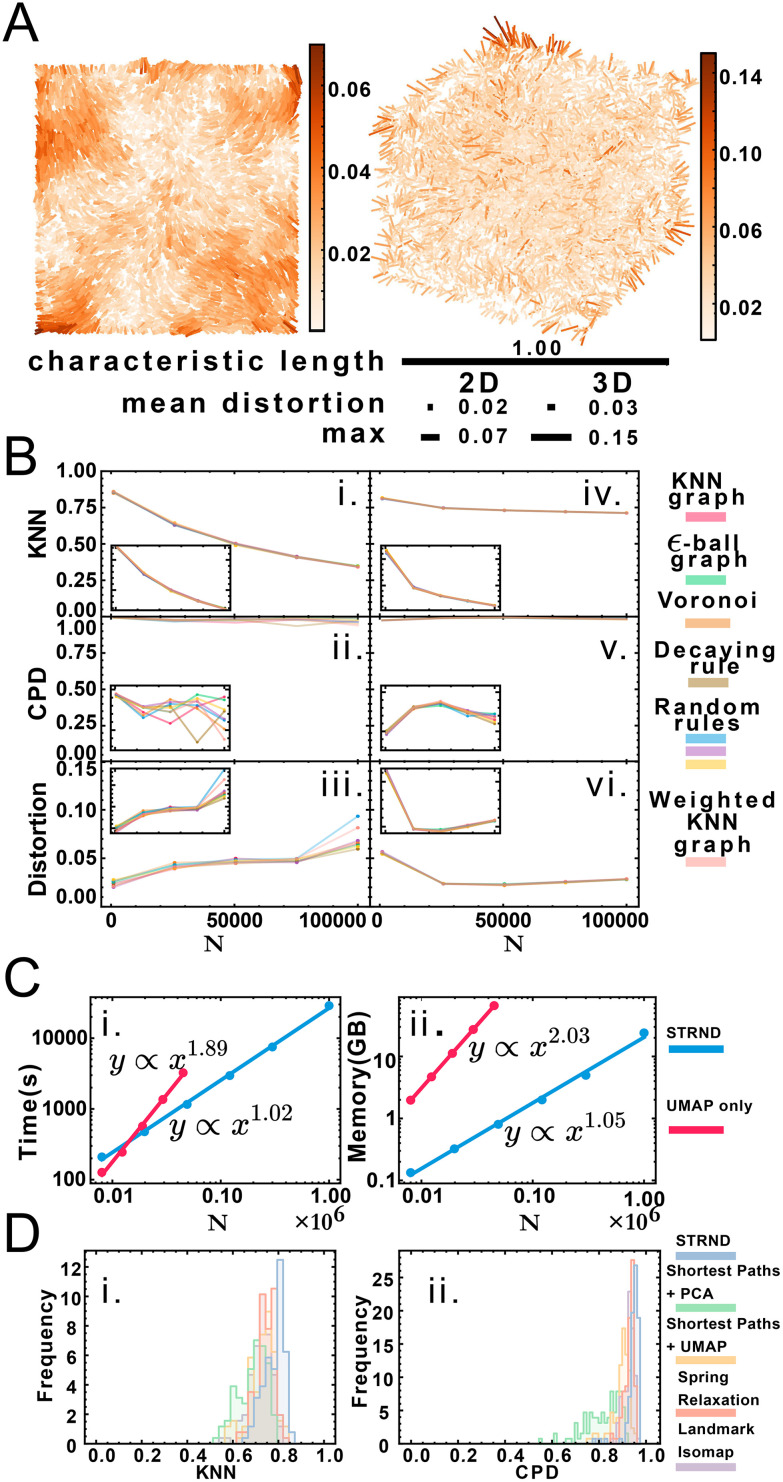
(A) Distortion map for the 2D and 3D cases on left and right respectively. Line segments connect original and reconstructed points and are colored according to the displacement (distortion) between them. Relative scale bars for the mean distortion, the maximum distortion and the characteristic length are shown underneath. (B) Quality metric dependence with proximity graph type and system size for 2D and 3D respectively: i. and iv. Local quality metric, ii. and v. Global quality metric, iii. and vi. mean distortion. Inset: reduced range to better appreciate changes in accuracy. (C) Computational complexity for the staged approach (blue) and the nonstaged approach (red) for time complexity (i) and space complexity (ii). (D) Comparison of recovery method robustness to random proximity rules in terms of KNN quality (i) and CPD quality (ii).

We obtained accurate reconstructions from both weighted and binary unweighted designs (Fig. 3B), which is noteworthy as the unweighted designs store less information than their distance-weighted counterparts. This would seem to support the validity of setups that only record whether an interaction happened or not (binary design) *versus* setups that record a measure of the distance between points (weighted design).

Node embedding significantly improves computational complexity (Fig. 3C). We compare STRND to direct manifold learning alone using a shortest-path distance matrix. While this approach can also reconstruct 2 and 3-dimensional images, its computational complexity becomes prohibitive for a large number of points, both time-wise and memory-wise. STRND addresses computational complexity by compressing the adjacency matrix using the random walk-based structural discovery step. Subsequent manifold learning becomes less resource-consuming, dealing with a *D*-dimensional vector instead of an *N*-dimensional vector (where *D* ≪ *N*). Whereas using only UMAP exhibits near-quadratic empirical scaling in both time and memory, STRND has near-linear complexity. This should enable large, fast reconstructions. Reconstructing a *N* = 10^6^ image using only UMAP would take years, and reconstructing the same image using the staged approach took eight hours. Moreover, we compare STRND to other approaches (discussed in ESI section 3.B†) to examine robustness. Fig. 3D shows that STRND has a slightly superior performance and little variation in regards to both local and global quality metrics.

Realizing the promise of imaging-by-sequencing will require robust, scalable reconstruction strategies. The method presented here addresses robustness to uncertainty in network generation mechanisms, however the field will also need tools for dealing with systematic variations in network structure as these might arise in biological imaging scenarios, *e.g.* anomalously high or low density regions. The problem of scalability will also need to be continuously addressed, as falling sequencing prices enabling greater experiment throughput will push the demand for computational efficiency. Finally, in this work we made use of quality metrics that compare reconstructed results to those of simulated ground truth data. However, it will be important to develop quality metrics that may be used without ground-truth knowledge as will be the case in experimental contexts. Our code may be accessed at: https://github.com/DavidFernandezBonet/ImageRecovery.

## Author contributions

DFB implemented the algorithms, characterization, and computational exploration in the study. DFB and ITH conceived the study and wrote the manuscript.

## Conflicts of interest

There are no conflicts to declare.

## Supplementary Material

NR-015-D2NR05435C-s001
