# Supplementary material for: Image recovery from unknown network mechanisms for DNA sequencing-based microscopy
Source: Nanoscale. 2023 Apr 14;15(18):8153–7. doi: 10.1039/d2nr05435c (PMC10173253; doi:10.1039/d2nr05435c)
Supplement: NR-015-D2NR05435C-s001 [file NR-015-D2NR05435C-s001.pdf]

This journal is © The Royal Society of Chemistry 2023

Supplementary Information

1 Extended background

1.A Characteristic connection patterns in imaging-by-sequencing

Imaging-by-sequencing is an emerging field whereby molecular networks and sequenced-based information are used instead of classical optics in order to convey spatial relationships between molecules. While there are a handful of strategies that have been proposed, a common aspect of these techniques is the use of network formation and spatial reconstruction from network information. The methods differ in their respective experimental approaches and consequently in the types of networks that they form, their basic structural patterns at the local level, as well as their global architecture. In this section, we attempt to organize these techniques and describe in greater detail, from a network perspective, the defining characteristics of each of these approaches and how this impacts the reconstruction strategy.

1.A.1 Parochial versus global connectivity pattern

One network class are those in which nodes exhibit a "parochial" connection pattern, i.e. nodes are locally confined and connections with other nodes are limited to those that are immediately adjacent or in very close proximity. These networks, like random geometric graphs or meshes, exhibit high homogeneity and isotropy - with a relatively constant degree distribution and an absence of any hubs or hierarchical structure. We proposed[1] that this could be the case for surface-grown DNA clusters or polonies, since the depletion of available primer sites by neighboring polonies would restrict growth, forming a Delaunay triangulation-type (or Voronoi tessellation) network. Another example of a network pattern restricted to local adjacency are the autocycling proximity recorder networks described by Yin and colleagues[2]. Proximity recorders have a fixed radius of interaction, suggesting that their network pattern would exhibit connections with a strict upper limit on connection probability. In principle, proximity recorders should also produce a relatively complete record of connections within the critical radius, suggesting that either the epsilon-ball connection pattern or the KNN graph would be an appropriate way to model this highly local pattern. Local confinement would also likely be the case for boundary-sharing cell networks such as[3] and iterative proximity ligation networks[4].

An alternative class of network patterns are those in which nodes are "promiscuous", or permitted to share a connection with nodes that are more spatially distant. These networks resemble a fully connected graph, but nevertheless exhibit isotropy and homogeneity albeit with a much higher average node degree. This could manifest in a limited sense as a relaxation of the conditions in the locally confined network patterns, where a node, rather than being strictly confined to its nearest neighbors or boundary-sharing neighbors, may in some cases form a connection with its second or third nearest neighbors. At the other end of the spectrum are network patterns with a high degree of long range global connectivity. This is the case in[5] and[6] where connections between a particular node may occur with any or most of counterpart nodes located throughout the global space. Likewise, a related format has been suggested in the case of GPS networks, where long range connections to each of multiple reference satellite nodes can occur with target nodes residing in the sample[7].

1.A.2 Binary unweighted networks versus networks with weighted edges

Spatial graphs are built from a set of points located in a space with a particular metric, most commonly Euclidean. In this work, we studied several ways to build such graphs by establishing proximity rules. This resulted in unweighted graphs because it captured a binary type of information: while edges linking nodes indicate neighborhood, absence of edges indicates absence of neighborhood (Fig. S1 A). As explored in this work, there are several ways to define neighborhood. For example, the Heaviside step function $f(\text{dist}) = H(\text{threshold})$ can be used to return 1 for nodes that are within a certain threshold distance and 0 otherwise (edge absence).

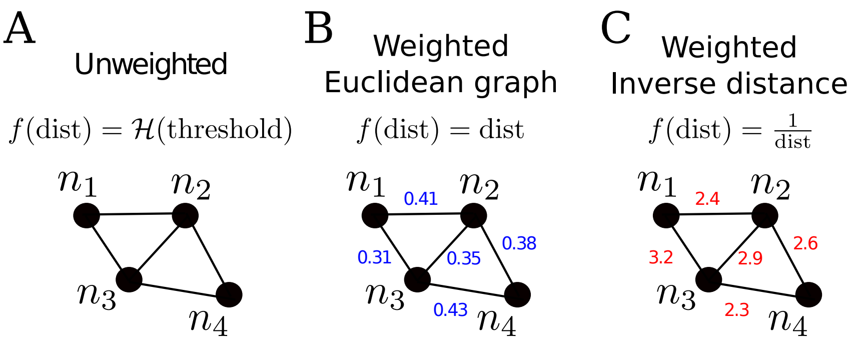

Fig. S1 A. Unweighted spatial graph. An edge exists if two nodes are within a certain threshold distance. B. Euclidean spatial graph. Edges are weighted using the pairwise distance between nodes. C. Weighted graph using the inverse distance. The closer a pair of nodes are, the greater their weight is.

However, edges can take values different from 0 and 1, therefore providing an additional layer of information. For instance, Euclidean graphs (Fig. S1 B) consist of spatial graphs where edges are weighted with the pairwise distance between nodes. Similarly, one can construct graphs where edges are weighted with a monotonically decreasing function with distance such as the inverse distance (Fig. S1 C). This case can model experiments where higher numbers are associated with closer distances. An example is the count of interactions between two molecules: the closer they are, the more interactions.

Weighted cases are of special interest to the Imaging-by-sequencing field if experiments are designed to quantize proximity beyond a binary manner. For example, DNA Microscopy by amplicon diffusion [5] leverages the fact that there is a relation between expected diffusion encounters and distance between molecules. Therefore, it is possible to weigh edges by counting the number of encounters. In this case, similarly to Fig. S1C, the closer a pair of molecules are, the greater the weight.

1.B Dimensionality reduction

The imaging-by-sequencing computational problem is one in which a high dimensionality dataset, e.g. an adjacency matrix of connections or a matrix of pairwise node-to-node distances, is used as input to recover the original spatial locations of the points that gave rise to proximity-dependent network topology. This progression from a large matrix to a small Cartesian coordinate vector thus falls under the umbrella of dimensionality reduction tasks, where the goal is to take a high-dimensional data set and map it onto a lower-dimensional space while preserving properties of interest in the source data. Dimensionality reduction techniques can be roughly broken down into linear and nonlinear categories:

1.B.1 Linear projection methods

Linear projection techniques, including principal component analysis (PCA), linear discriminant analysis (LDA), and random projections, aim to find a linear combination of input features that maximizes variance in the data. Projection methods assume that data points in a data set are correlated, and that the most important features are ones that explain the variance in the data. If the data points are sampled from a low dimensional manifold and embedded in a high dimensional space, as in the case of a pairwise inverse distance matrix or matrix of shortest path graph distances [1], then the directions with the greatest variance in the data points may align with those that preserve pairwise distances or the local neighborhood structure of the data.

1.B.2 Nonlinear methods

Nonlinear techniques can capture non-linear relationships in the input matrix and can be more effective at preserving important features of the original data. These include manifold learning techniques like Isomap [8], locally linear embedding (LLE) [9], Laplacian Eigenmaps [10], and node embedding methods. These techniques are based on the idea that high dimensional data lies on or close to a lower-dimensional manifold and use non-linear functions to discover a representation of the data that preserves the geometry of the data by maintaining pairwise distances between points.

2 Methods

Of particular interest to us are the node embedding methods, including DeepWalk [11], Large-scale Information Network Embedding (LINE) [12], PyMDE [13], and Node2vec [14]. These are methods that learn a low-dimensional representation of the nodes in a graph. By pairing this technique with a manifold learning technique, we are able to set up an autoencoder format whereby a high-dimensional data set, such as the graph adjacency matrix, is first reduced to an intermediate medium-dimensionality encoding using node embedding and subsequently decoded by using manifold learning or other dimensionality reduction to achieve the final low-dimensionality embedding comparable to the original points.

2.A Node2Vec

Node2Vec is a node embedding technique that learns graph structural features by performing biased random walks and embedding nodes into low-medium-dimensional vectors. It effectively compresses the information of the adjacency matrix into a $N \times D$ matrix. Storing information in such a way improves the overall computational complexity for the subsequent manifold learning and the robustness of our approach. The node embedding is optimized by maximizing the log-likelihood of observing a node given a particular random walk context. In short, several random walks are performed starting at each node while storing every visited node. The sequence of nodes visited is used to train a skip-gram model. This results in feature vectors that are low dimensional representations of each node. The embedding algorithm optimizes for an objective function that maximizes neighborhood proximity likelihood, and thus feature vectors populated with similar values are likely to reflect nodes that are close in spatial proximity. Negative sampling is used to reduce the optimization's computational complexity.

2.B UMAP

Uniform Manifold Approximation and Projection (UMAP) [15] is a nonlinear manifold learning technique that can be used to reduce the number of dimensions in a data set while preserving important features. UMAP is designed to be fast and efficient and furthermore

is focused on preserving both global and local relationships between points by minimizing the distance between the original high-dimensionality data and its low-dimensionality embedding. Because of this, it is a more desirable method when one wishes to preserve spatial proximity information accurately. It is worth noting that UMAP can directly recover images without requiring a previous node embedding step, as done in Fig.3 C. A dense version of the adjacency matrix can be obtained by computing the shortest-path distance between every node, where d_{ij} is the shortest-path distance between node i and node j. The result is a $N \times N$ distance matrix, assigning N-dimensional vectors to each node. Here, the linear relation between Euclidean distance and graph shortest-path distance is exploited to obtain high-dimensional vectors that preserve Euclidean geometric information.

2.C Hyperparameters

We use the values shown in Table S1 as default hyperparameters when using Node2Vec and UMAP, having found them to be a good compromise between reconstruction accuracy and low computational complexity, as will be discussed below from studying Tables S2-S6.

Table S1 Image reconstruction hyperparameters. Default value and definition

Parameter	Default value	Definition
neighbors	15	[UMAP] average number of neighbors per node
embedded dimension	32	[Node2Vec] each node is mapped into a vector of that many dimensions
1/q	1	[Node2Vec] the higher the more Breadth-First-Search alike random walks
1/p	1	[Node2Vec] the higher the more Depth-First-Search alike random walks
random walk length	10	[Node2Vec] number of visited nodes in each random walk

We also investigate the influence on the local and global quality metric over deviations from the default parameters (Fig. S2).

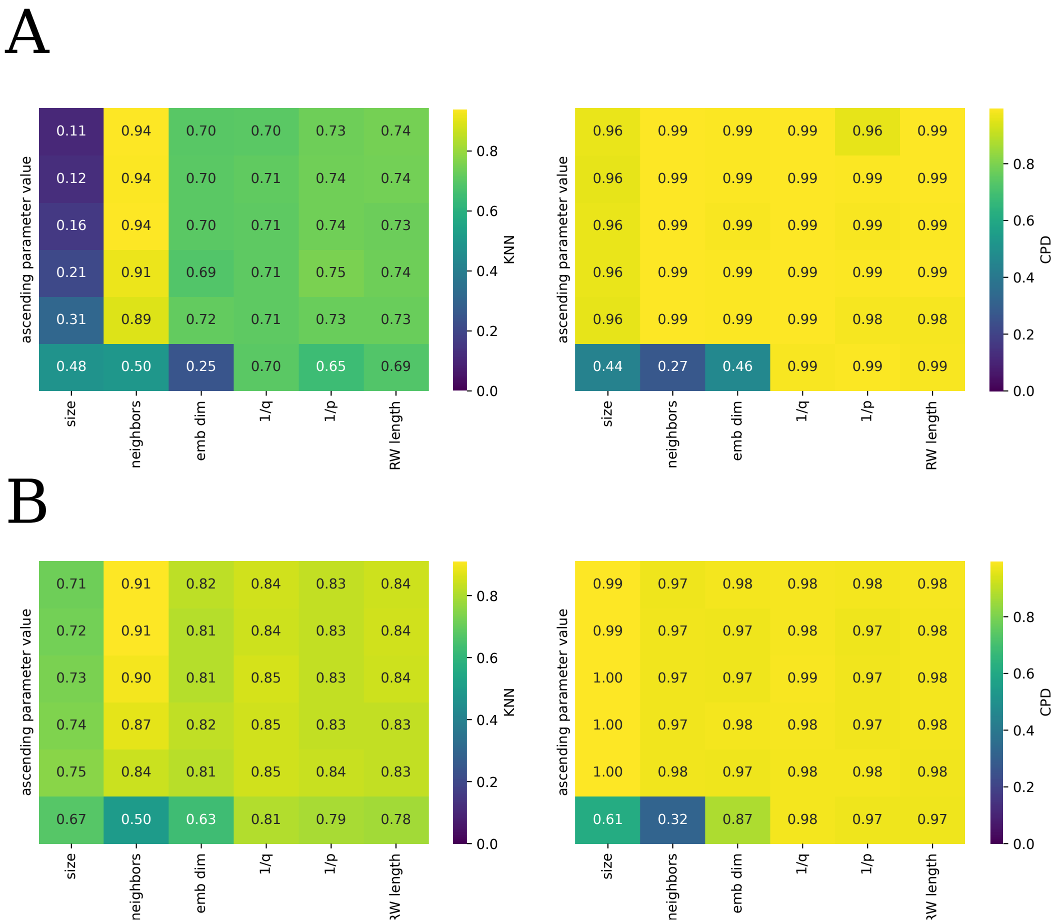

Fig. S2 Local and global quality metric dependence with hyperparameters in A. 2 dimensions and B. 3 dimensions.

2.C.1 Grid Search study

We evaluated the accuracy and computational complexity (time, memory) for different hyperparameter values to establish default values (Table S1). Evaluations were done for a system size of $N = 5000$ and can be found in Tables S2-S6.

As can be seen in Table S2, a low embedded dimension results in poor accuracy. However, after a dimensionality threshold of 16, accuracy values remain similar while computational complexity increases for this system size.

As can be seen in Table S3, an increase in the estimated number of neighbors has, in general, a positive effect on the accuracy scores. However, accuracy can decrease if such a value is too large (from 512 to 2048). We conclude that a value of the order of magnitude ~ 10 produces accurate enough results while its space complexity is low for this system size.

Both $1/q$ and $1/p$ variations in hyperparameter value are not particularly impactful on accuracy (Tables S4, S5), although higher values contribute to a better local quality metric. Furthermore, choosing a value of the order of magnitude below or above 1 contributes to a higher time complexity. We suggest keeping such hyperparameters at value 1. This is equivalent to pure random walks with no bias towards Breadth-First Search or Depth-First Search strategies.

Finally, the random walk length has a positive effect on the local quality metric KNN score but also leads to an increase regarding time and space complexity (Table S6). We consider the trade-off to be adequate for a random walk length of the order of 10, although high system sizes might require greater values. This is further explored in section 2.C.2.

Table S2 Accuracy and computational complexity for different D embedded dimension values, where $N \times D$ is the matrix obtained after applying structural discovery.

**embedded dimension**	KNN	CPD	Distortion	Time (s)	Memory (MB)
4	0.2869	0.3442	0.5251	116.6229	98.2401
16	0.8359	0.9974	0.0142	115.2382	98.2451
32	0.8362	0.9978	0.0133	114.4272	98.2748
128	0.8347	0.9978	0.0133	117.9136	98.2391
512	0.8359	0.9973	0.0156	126.8496	183.5303
2048	0.8335	0.9973	0.0148	161.7088	594.6994

Table S3 Accuracy and computational complexity for several estimated numbers of neighbors (UMAP parameter).

**number of neighbors**	KNN	CPD	Distortion	Time (s)	Memory (MB)
4	0.4518	0.8566	0.1552	111.4169	98.2939
16	0.8387	0.9979	0.0126	114.3205	98.2579
32	0.8551	0.9981	0.0113	116.8359	99.2875
128	0.9223	0.9977	0.0136	130.1769	451.4392
512	0.9305	0.9928	0.0219	137.0709	535.6371
2048	0.8554	0.8967	0.1079	171.362	927.4699

Table S4 Accuracy and computational complexity for different $1/q$ weights.

**1/q**	KNN	CPD	Distortion	Time (s)	Memory (MB)
0.001	0.8141	0.9978	0.0129	1401.4252	98.2432
0.01	0.818	0.9968	0.0164	1399.4103	98.2473
0.1	0.8147	0.9969	0.0153	1404.5331	98.2877
1	0.8371	0.9969	0.0162	115.1361	98.2731
10	0.834	0.9961	0.0152	1414.214	98.2421
100	0.8405	0.9973	0.0181	1407.2681	98.2517

Table S5 Accuracy and computational complexity for different $1/p$ weights.

**1/p**	KNN	CPD	Distortion	Time (s)	Memory (MB)
0.001	0.8372	0.998	0.013	1423.1641	98.2725
0.01	0.8342	0.9984	0.0137	1415.0215	98.2461
0.1	0.8349	0.9983	0.0122	1424.8976	98.311
1	0.8363	0.9982	0.0127	115.8922	98.2497
10	0.8487	0.9976	0.0127	1410.4853	98.2243
100	0.8646	0.9979	0.014	1413.0953	98.2343

Table S6 Accuracy and computational complexity for different random walk lengths

**random walk length**	KNN	CPD	Distortion	Time (s)	Memory (MB)
3	0.7725	0.9927	0.0296	45.9482	59.7399
6	0.8184	0.9941	0.0193	74.7885	72.0304
12	0.8440	0.9981	0.0136	136.0008	111.3782
24	0.8526	0.9978	0.0125	257.4416	190.0895
49	0.8624	0.9974	0.0119	561.9080	354.1267
100	0.8708	0.9980	0.0115	1102.4371	688.7350

2.C.2 Accuracy impact of the Embedded Dimension and Random Walk Length

We observe that accuracy results are particularly sensitive to the length of the random walks and the choice of embedded dimension (Tables S2, S6), and elaborate further in this subsection.

Regarding the embedded dimension, an educated assumption is to consider that the best accuracy is achieved when the embedded dimension is equal to the number of nodes in the network. However, an optimal dimension can be found by comparing different embedding dimensions[16]. Because of the low accuracy resulting from using an embedding dimension of 4 (Table S2), we find empirically that the optimal embedding dimension has to be greater than 2 or 3, the dimension of the original positions. Further empirical results supporting this can be found in Tables S8-S13, where Node2Vec was implemented without a subsequent dimensionality reduction, i.e., directly embedding in 2 or 3 dimensions respectively. The accuracy results are low in comparison to other methods, suggesting that choosing a low embedding dimension does not provide good performance regarding image reconstruction accuracy.

A possible interpretation of why this happens is that the information encoded in the feature vectors is complex enough that it has to be represented through more than 2 or 3 coordinates. This is not unreasonable as the information encoded in the spatial graph is related to the neighborhood of nodes rather than its exact Euclidean distance. This means that the underlying dimension of the graph is likely to be higher than the underlying dimension of the original points. For instance, if 4 points are originally close to each other they will form a K_4 graph (unweighted complete graph with size 4). However, it can be proven that a K_4 graph cannot be perfectly embedded in 2D while exactly preserving graph distances. Therefore, the network structure is in general more accurately depicted through higher embedding dimensions. After the features are encoded in D-dimensional vectors, it is possible to use a manifold learning procedure such as UMAP under the assumption that similar feature vectors correspond to points that are close in the original space.

Moreover, Fig.2 B shows a notable decline in performance with a system size increase. Consequently, properly choosing hyperparameter values with regard to the system size might be a key aspect of preserving accurate results. We investigate the accuracy impact of independently changing the embedding dimension and the random walk length with different system sizes (Fig.S3). In general, increasing hyperparameter values led to an increase in accuracy. For the local KNN metric we set a standard accuracy threshold of 0.8, dividing the grid points that reached this standard from the ones that did not (Fig.S3 A). A similar procedure is done in (Fig.S3 B) with an accuracy standard of 0.98. This reveals that the embedding dimension and the random walk length have to be raised in order to keep the same standards, with a few exceptions for the global metric and the random walk length.

We further study the behavior of changing both the embedding dimension and the random walk length in Fig.S4 by setting the same accuracy thresholds. For the local KNN quality metric, an increase in size requires raising both the value of the embedding dimension and of the random walk length to preserve the same accuracy standards. Conversely, a lower random walk length and a higher embedding dimension are required to preserve the same global CPD quality metric. This suggests, on the one hand, that higher system sizes require raising the embedding dimension to be as accurate as lower system sizes. On the other hand, the random walk length acts as a trade-off between the local and global structure, as raising it can improve the local quality metric but also worsen the global quality metric when the embedding dimension is high enough.

2.C.3 Modeling Accuracy with a non-linear squares fit

In this section, we study STRND's performance to understand better how can we can maintain a given accuracy standard by changing hyperparameters. In particular, we want to find the accuracy function $A(x,y,z)$ that depends on three variables: embedded dimension, random walk length and system size. For the sake of simplicity, they will be referred to as x,y,z respectively. We chose these parameters because they have a considerable impact on accuracy scores (in particular, the KNN local metric) as can be observed from Tables S2, S6 and Fig.2 B. While an increase in embedded dimension and random walk length have a positive impact on accuracy, an increase in system size has been observed to have a negative impact.

An educated guess for the accuracy function is to assume that each variable has a logarithmic impact and are independent from one another:

$$A(x,y,z) = \log(a) + b\log(x) + c\log(y) + d\log(z)$$

where A is the accuracy score, a,b,c,d are parameters and x,y,z are the embedded dimension, the random walk length and the system size respectively. In order to find the parameters a,b,c,d and obtain the corresponding fit, we use data containing several combinations of x, y, z and their associated accuracy. In particular, the embedded dimension values range from 4 to 32, the random walk length also from 4 to 32 and the system size from 1000 to 18000. The fit we find will be bounded to such values, meaning that predictions below and over them might not be valid. Furthermore, the assumptions that x,y,z are not coupled and that they have a logarithmic impact are not necessarily true. Nonetheless, we find a fit using the non-linear least squares method and test it. As seen in Fig.S5 A, the values predicted by the fit and the ground truth do not have a linear correspondence, possibly to the nature of the assumptions made to find the fit. However, they have a logistic correspondence, both for the KNN and for the CPD quality metric. We decide to perform a second fit, this time a logistic fit with its corresponding parameters: L, k, x_0. As seen in Fig.S5 B, the predicted and ground truth accuracy scores now have a linear relationship with noise. The accuracy equation becomes:

$$A(x,y,z) = \frac{L}{1 + \exp^{kx_0} \exp^{-k[\log(a) + b\log(x) + c\log(y) + d\log(z)]}}$$

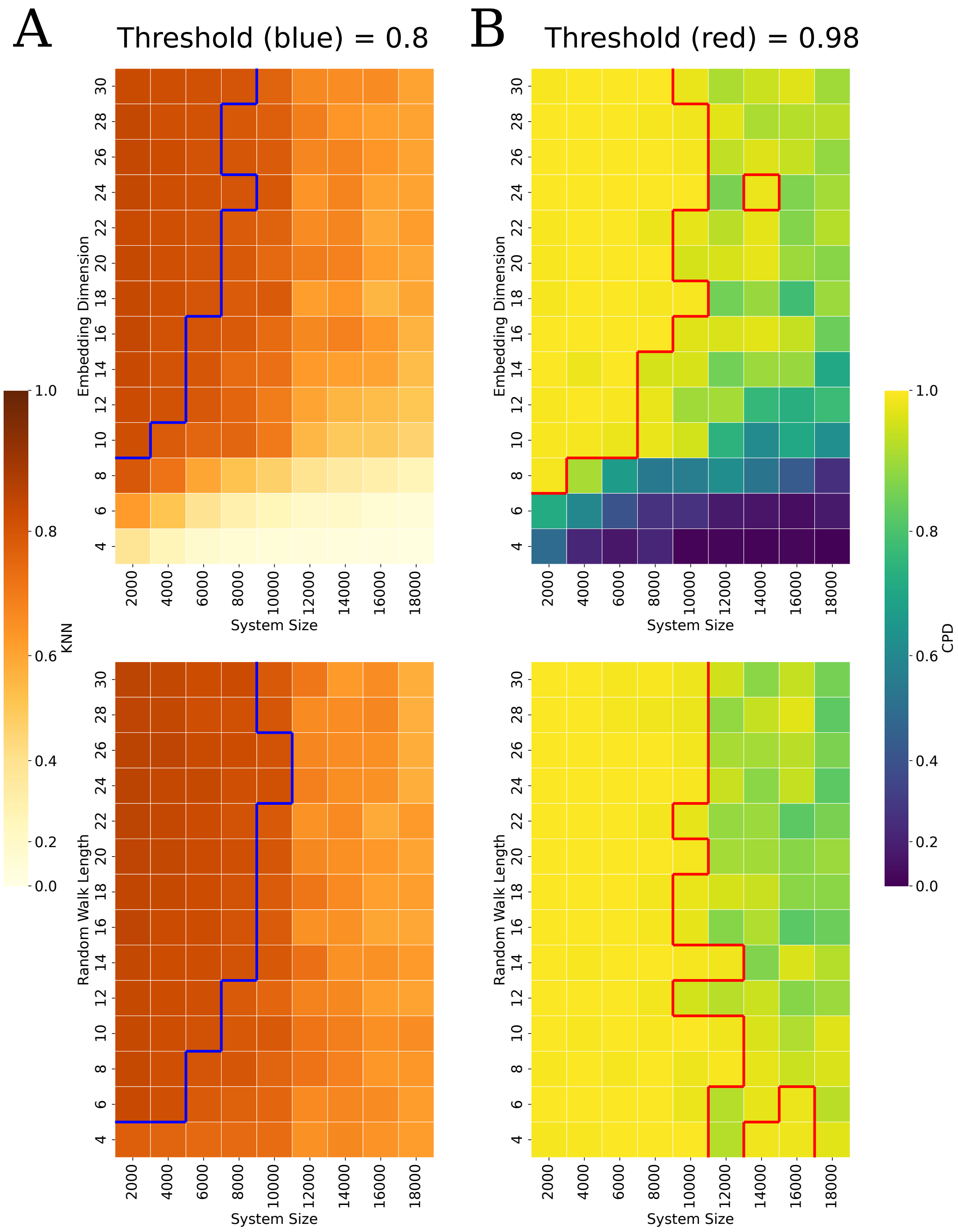

Fig. S3 Accuracy dependence with system size, embedding dimension and random walk length. A. Local KNN quality metric, blue isocurve corresponding to KNN=0.8. B. Global CPD quality metric, red isoline corresponding to CPD=0.98.

This equation can predict accuracy in an approximate manner for a bounded range of its variables x, y, and z. For example, it is possible to set an accuracy preservation condition, $A_1(x_1, y_1, z_1) = A_2(x_2, y_2, z_2)$ and obtain:

$$x_1^{-kb} - x_2^{-kb} + y_1^{-kc} - y_2^{-kc} = z_2^{-kd} - z_1^{-kd}$$

This is particularly useful for the task of choosing the embedded dimension x_2 and the random walk length y_2 that preserves accuracy when changing to a system size z_2, given a previous accuracy observation with variables x_1, y_1, z_2. The results obtained with this approach are an approximation. However, it can provide educated guesses that may lead to faster results when compared to solving the task of accuracy preservation manually.

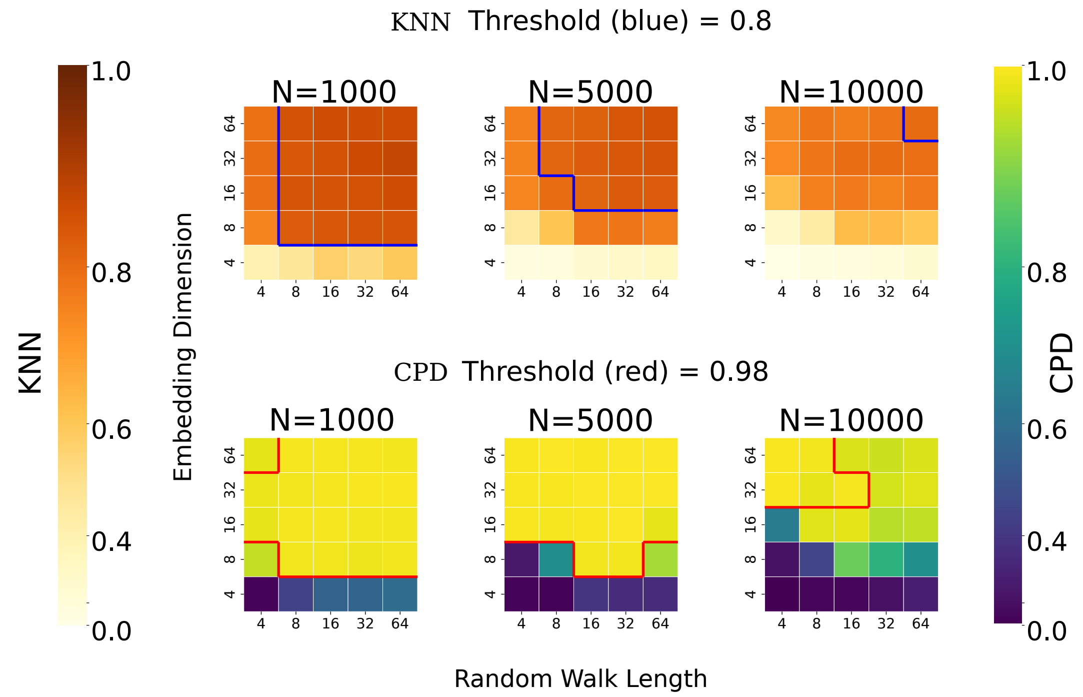

Fig. S4 Accuracy dependence with changes in both embedding dimension and random walk length for three different system sizes: N=1000, N=5000 and N=5000.

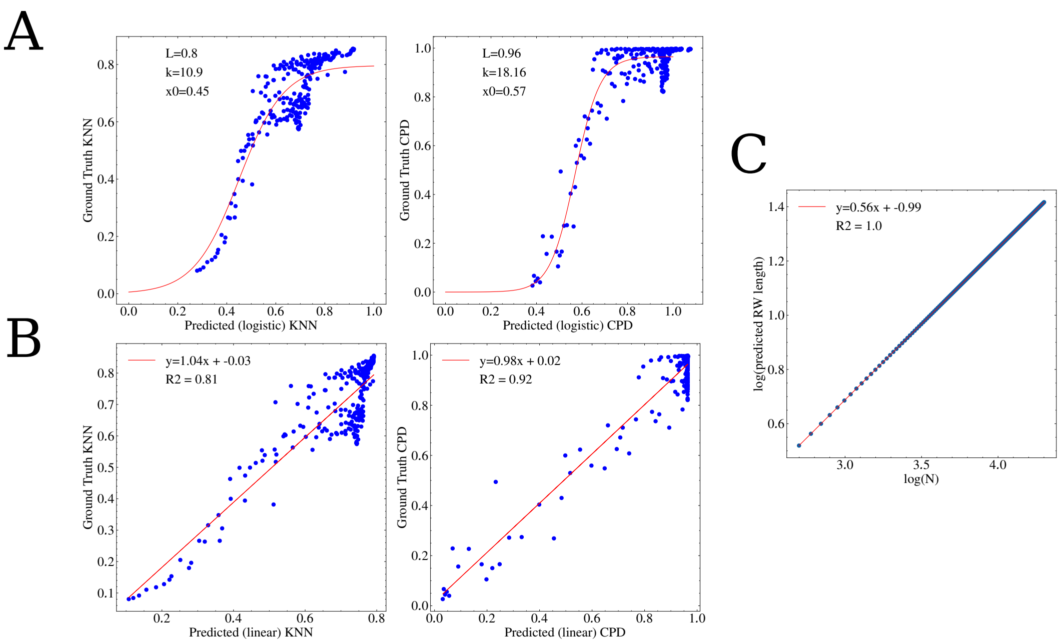

Fig. S5 A. Ground truth and predicted accuracy (KNN and CPD) for a logarithmic fit. The data can be modeled with a further logistic fit with parameters L, k and x_0. B. Ground truth and predicted accuracy (KNN and CPD) after applying the logistic fit. The result is a (noisy) linear correspondence between ground truth and prediction values. C. Predicted random walk dependence with size in order to preserve predicted accuracy.

2.C.4 Computational complexity of constant accuracy reconstructions

In the previous section, we built an accuracy predictor that predicts performance given certain reconstruction hyperparameters. In this section, we investigate the computational impact of using the values suggested by such a predictor. In particular, we focus on the problem of finding configurations that preserve accuracy given size variations for the 2D case. We use the local KNN quality metric as an accuracy measure and the random walk length as the variable that can raise computational complexity. The reason we choose random walk length and not embedded dimension is that changes in random walk length will lead to different empirical complexities for low values (ranging from 4 to 32), which is a behavior that is not observed for the embedded dimension (Tables S2, S6).

Fig.S5 C shows the random walk length value that is suggested by the predictor given a certain system size in order to achieve an accuracy standard of 0.7. We find that the predictor suggests that for a size increase $Z = \frac{z_2}{z_1}$, the random walk length increase should be $Y = Z^{0.56}$. We reconstructed images with 7 different sizes ranging from 500 to 15000 following this random walk length behavior, which resulted in random walk lengths ranging from 3 to 19. Furthermore, we performed 50 reconstructions for each size in order to increase the statistical validity of the results. We annotated the KNN accuracy, time and space complexity and computed their average value for every size. Fig.S6 A shows that the empirical time complexity that preserves accuracy is of order $O(N^{1.08})$, which is greater than the complexity we found for a constant random walk length in Fig.2 C ($O(N^{1.02})$). This supports the intuition that in order to preserve accuracy the time complexity must increase. Here we present a quantitative measure for such an increase. However, Fig.S6 shows that an increased random walk length presents a space complexity is $O(N^{1.04})$, while a constant random walk length presented a complexity of $O(N^{1.05})$. Therefore, peak memory values are affected by the system size rather than random walk length, at least for values ranging from 3 to 19 and sizes from 500 to 15000. Fig.S6 C shows the mean accuracy values for each size. The reconstructed images had mean accuracy values ranging from 0.68 to 0.81, which are close to the accuracy standard 0.7 used to choose the random walk length hyperparameter.

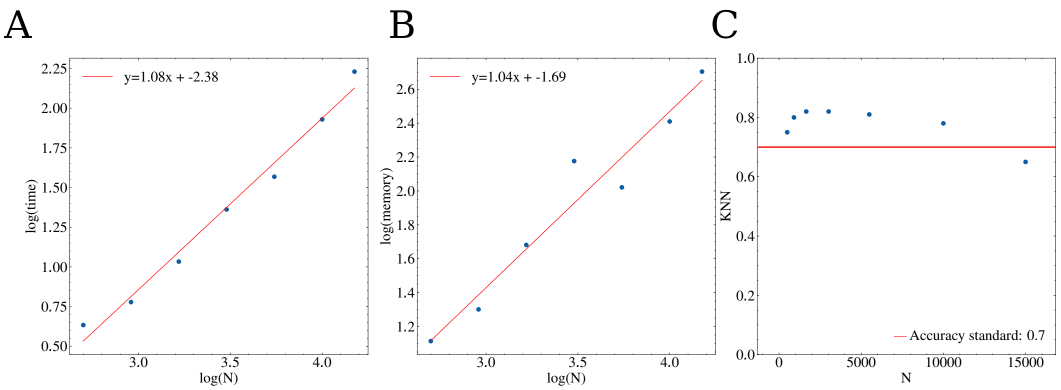

Fig. S6 Reconstructed images with random walk length chosen according to system size to preserve accuracy values. A. Empirical time complexity. B. Empirical space complexity. C. Local KNN metric for different sizes and the accuracy standard used to choose random walk length.

2.D Quality Metrics

2.D.1 The local and global structure

Local structure is preserved when points that are close to each other in the original image are also close in the reconstructed image. In this work, local structure is investigated through the KNN quality metric, which evaluates the fraction of neighbors preserved in the reconstruction. Conversely, global structure is preserved when points that are separated far apart in the original image are equally far in the reconstructed image. In this case, the quantity to be preserved is separation distance, and it can be investigated by computing the correlation between pairwise distances.

2.D.2 KNN

The KNN quality metric can be formally defined as

$$\text{KNN} = \frac{1}{N}\sum_{i}^{N}\sum_{j}^{k}\frac{q_{ij}}{k}, \qquad q_{ij} = \begin{cases} 1, & \text{if } i, j \text{ neighbors in both original and reconstructed} \\ 0, & \text{otherwise.} \end{cases} \tag{S1}$$

Here, N is the total number of elements and k is the number of considered neighbors. We used $k = 15$ for quality metrics presented in this manuscript. Effectively, KNN computes the fraction between original and reconstructed neighbors (inner summation) for every node (outer summation) and averages it over the total number of elements.

2.D.3 CPD

The CPD quality metric is the Pearson correlation between original and reconstructed pairwise distances. As opposed to KNN, CPD measures concern not only neighboring points but also distant points. The motivation to measure correlation is to evaluate distance preservation. If pairwise distances in the original image are linearly correlated with pairwise distances in the reconstructed image, then the reconstructed image is equivalent to the original image up to an affine transformation. Dealing with scalability often require workarounds: for N points, there are $\frac{N(N-1)}{2} \propto N^2$ pairwise distances, which can be computationally prohibitive for large N. The adopted solution is to randomly sample $N = 1000$ points and compute their 499500 pairwise distances for the original and reconstructed points, to later obtain a representative Pearson correlation.

2.D.4 Distortion

Point cloud alignment is necessary to compute distortion, which is defined as the distance between every original and reconstructed point pair. Reconstructed points need to be aligned with the original points because scale, rotation, translation, or chirality are not necessarily preserved. Consequently, point cloud alignment consists in finding the affine transformation such that distortion between original and reconstructed points is minimized. To find such a transformation, we apply the coherent point drift algorithm[17].

Nonetheless, coherent point drift is usually not enough to successfully align reconstructed points because for two reasons. First, it does not consider reflection transformations which account for chirality corrections. Second, if the original and reconstructed point clouds are initially far apart, the algorithm might not converge in the optimal transformation. To solve this, we first perform a set of transformations over the reconstructed points involving scaling obtained by computing the correlation between pairwise distances, point-to-point translation and rotation, and the set of coordinate reflections that minimize distortion.

2.D.5 Quality metric variation with proximity graph

To examine the robustness of STRND we studied the quantitative quality metric variation to proximity graph change. The results are presented in Table S7.

Table S7 Mean and maximum quality metric variation over the different proximity graphs, for the 2D and 3D case.

	Mean variation - 2D	Max variation - 2D	Mean variation - 3D	Max variation - 3D
KNN	0.011	0.015	0.002	0.007
CPD	0.032	0.058	0.003	0.006
Distortion	0.011	0.033	0.001	0.002

3 Comparative Study

3.A Other approaches

As there are multiple possible image recovery routes, different dimensionality reduction techniques, and combination pipelines, we compared a selection of approaches with STRND in terms of quality metrics, computational complexity and robustness to network patterns.

3.A.1 Shortest-path distance matrix

Shortest-path distance matrices are obtained by applying a Breadth-First-Search algorithm to a sparse adjacency matrix. The result is a densely-populated $N \times N$ matrix where the element N_{ij} contains the shortest-path distance between node i and node j. Such distance matrix can be reduced to a $N \times 2$ or $N \times 3$ matrix using e.g. PCA or UMAP (parameters in Table S1), as shown in section 3.B.

3.A.2 Spring relaxation

Force-directed approaches are used as a means to layout graphs in aesthetically pleasing ways. They are inspired by physical systems: nodes in a graph are considered particles that attract each other if they are close and repel otherwise. In particular, we implement the Fruchterman-Reingold algorithm[18], where edges between nodes can be considered physical springs storing elastic potential energy. The goal is to find the set of node positions that reaches equilibrium, i.e., to find the global minimum of the energy function. In particular, we use the Networkx[19] implementation with 300 iterations to ensure convergence.

3.A.3 Landmark Isomap

Landmark Isomap was implemented by randomly sampling D landmark nodes, computing the squared shortest-path distance between every landmark and every node in the graph, and finally triangulating nodes positions through Multidimensional Scaling (MDS)[20]. The squared shortest-path computation results in a $N \times D$ matrix, as there are N nodes and D landmarks. Such computation is done by iterating the single-source Breadth-First-Search algorithm for every landmark. This approach is considerably better than computing the all-pairs shortest-path distance matrix in terms of empirical computational complexity, as it avoids computing a $N \times N$ matrix. The next step is to store the $D \times D$ squared distance matrix between all landmarks, as it is used to map the landmark nodes into the reconstructed space by applying MDS. Finally, the rest of the nodes are triangulated by solving an eigenvalue problem on the Moore-Penrose pseudo-inverse of the $D \times D$ landmark matrix[3]. As the default embedding dimension for STRND is 32, we also use $D = 32$ landmarks. Nonetheless, we study the accuracy impact of the number of landmarks in the section 3.B and in particular in Fig.S8.

3.A.4 DNA Microscopy by Amplicon Diffusion

Amplicon diffusion imaging is an image recovery approach tailored for a particular experimental setting that uses a weighted scheme[5]. It relies on molecular diffusion to gather proximity information. Proximity information can be extracted because there is a physical link between the expected diffusion encounters between molecules and their distance. It distinguishes from unweighted approaches which only gather binary proximity information regarding neighborhood, i.e., establishing if molecules are neighbors or not.

As a benchmark for the quality metrics used in this work, amplicon diffusion was simulated and reconstructed according to the recovery method from Weinstein and colleagues' code. The simulation was done for a system size of $N = 10000$ randomly positioned beacon and target molecules and 100 Unique Event Identifiers (UEI) per molecule[5]. A large enough number of UEI per molecule was chosen for a more accurate reconstruction. The total number of molecules is not the number of target (imaged) molecules, which is $N \approx 5000$ in this simulation. The original and recovered image through the amplicon diffusion algorithm (which produces its own weighted proximity graph) can be seen in Fig.S7 A and B, along with the respective quality metrics. The global CPD quality metric is of similar magnitude to STRND (Tables S8, S9, S10). Close inspection reveals the presence of outlier points that influence the KNN local quality metric. These outliers can also be seen in Fig.S7 C, the distortion resulting from aligning original and recovered points, and Fig.S7 D in the distribution of distortion values.

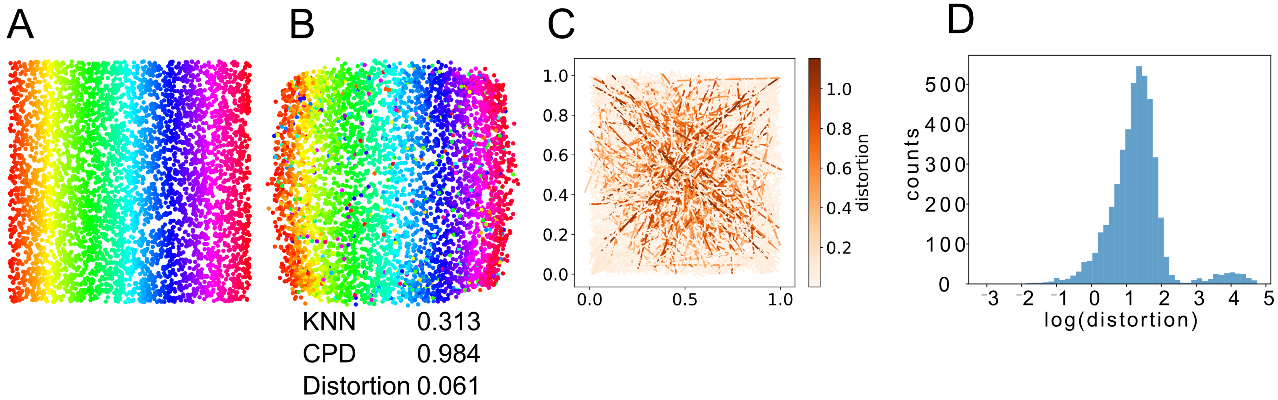

Fig. S7 Amplicon diffusion simulation results. (A) Original image, labeled with a color gradient according to the horizontal axis. (B) Reconstructed image and its corresponding quality metrics values. (C) Comparison between original and recovered points through a distortion plot. (D) Semi-logarithmic histogram for the distortion values.

3.B Benchmark comparison

The robustness of each approach is first studied by generating unbiased network pattern rules in Fig.3 D. Such rules were obtained from Gaussian mixture distributions with randomly selected parameters (10 individual distributions each). For every reconstruction method, 100 images were recovered with system size $N = 100$. To compute reconstruction accuracy we use the local KNN quality metric and the global CPD quality metric, abstaining from using point cloud alignment (distortion) as it is not guaranteed to find the optimal affine transformation when reconstruction accuracy is low.

Although a low system size enables the reconstruction of many images in a limited time, it may be subject to boundary effects. Therefore, we further study a system size of $N = 5000$ in Tables S8-S13. However, we restrict the network patterns to three representative scenarios for the 2D and 3D case: the KNN graph, the Delaunay graph and a graph with a (stochastically) decaying probability with distance. To compute the empirical computational complexity we evaluate the total time and peak memory used by the image recovery algorithm. Corresponding reconstructions from the benchmark are shown in Fig.S9.

Tables S8, S9, S10 contain the accuracy and computational complexity for three different proximity graphs for the 2D case, whereas Tables S11, S12, S13 are the 3D case equivalent.

Shortest Paths + PCA obtains satisfactory results for both the local and global quality metric in specific cases, e.g. for the KNN proximity graph (Tables S8, S11). PCA is reported to perform better in terms of global structure preservation than local[21] as was observed in most proximity graphs we tested. Second, Shortest Paths + UMAP slightly outperforms PCA in every case both for the local and global structure, except for the 3D Delaunay graph (Table S12) where the global quality metric is marginally lower.

Spring Relaxation stands out for its high empirical running time. Its worst-case computational complexity is quadratic with the number of nodes, $O(N^2 + E)$, where N is the number of nodes and E is the number of edges. Interestingly, spring relaxation performs better for the 3D case (Tables S11, S12, S13) than for the 2D case (Tables S8, S9, S10).

Table S8 Accuracy and computational complexity of different image recovery approaches for the 2D KNN graph.

**KNN graph – 2D**	KNN	CPD	Time (s)	Peak memory (MB)
Node2Vec Only	0.0401	0.1424	23.8055	82.3880
Shortest-Paths – PCA	0.7072	0.9724	36.8774	646.6104
Shortest-Paths – UMAP	0.7920	0.9968	45.9498	804.7638
Spring Relaxation	0.5871	0.5355	745.4615	35.2796
Landmark Isomap	0.7037	0.9966	1.9879	31.4567
STRND	0.8396	0.9981	135.6499	108.0467

Table S9 Accuracy and computational complexity of different image recovery approaches for the 2D Delaunay graph.

**Delaunay graph – 2D**	KNN	CPD	Time (s)	Peak memory (MB)
Node2Vec Only	0.0383	0.0651	23.8106	80.8552
Shortest-Paths – PCA	0.2620	0.2041	35.7504	632.8743
Shortest-Paths – UMAP	0.6186	0.9131	43.2034	790.9597
Spring Relaxation	0.3714	0.5750	724.4941	16.4635
Landmark Isomap	0.2659	0.1094	1.2401	14.7346
STRND	0.8355	0.9980	114.6659	98.2327

Table S10 Accuracy and computational complexity of different image recovery approaches for the 2D random-rule graph.

**Stochastic Decay graph – 2D**	KNN	CPD	Time (s)	Peak memory (MB)
Node2Vec Only	0.0433	0.1687	24.3959	82.2907
Shortest-Paths – PCA	0.5686	0.965	36.9282	646.0127
Shortest-Paths – UMAP	0.6399	0.9944	46.5864	812.982
Spring Relaxation	0.532	0.4891	723.3835	34.599
Landmark Isomap	0.5466	0.9957	1.9275	29.5123
STRND	0.8365	0.9984	115.244	98.2323

Table S11 Accuracy and computational complexity of different image recovery approaches for the 3D KNN graph.

**KNN graph – 3D**	KNN	CPD	Time (s)	Peak memory (MB)
Node2Vec Only	0.0984	0.2545	23.7324	82.4992
Shortest-Paths – PCA	0.7138	0.9704	39.3418	646.9862
Shortest-Paths – UMAP	0.7832	0.993	47.1005	804.7801
Spring Relaxation	0.7798	0.9723	748.0907	35.924
Landmark Isomap	0.6835	0.9845	2.0294	31.5816
STRND	0.7844	0.9896	115.9369	98.7001

Table S12 Accuracy and computational complexity of different image recovery approaches for the 3D Delaunay graph.

**Delaunay graph – 3D**	KNN	CPD	Time (s)	Peak memory (MB)
Node2Vec Only	0.1122	0.3181	23.9456	82.8388
Shortest-Paths – PCA	0.2937	0.4839	39.6028	646.6734
Shortest-Paths – UMAP	0.3794	0.4532	47.9258	805.2829
Spring Relaxation	0.5416	0.8926	757.3058	34.9591
Landmark Isomap	0.2733	0.4603	2.4268	34.3637
STRND	0.7838	0.9891	114.9723	98.6993

Table S13 Accuracy and computational complexity of different image recovery approaches for the 3D random-rule graph.

**Stochastic Decay graph – 3D**	KNN	CPD	Time (s)	Peak memory (MB)
Node2Vec Only	0.1127	0.3504	23.9255	82.5592
Shortest-Paths – PCA	0.5308	0.9487	38.7972	654.0787
Shortest-Paths – UMAP	0.6004	0.9853	49.7117	804.2844
Spring Relaxation	0.7042	0.9836	752.1702	34.7898
Landmark Isomap	0.4791	0.9758	2.009	31.0177
STRND	0.7824	0.9902	115.1676	98.6997

Similarly to the Shortest Paths + PCA approach, Landmark Isomap displays a high level of accuracy for the KNN proximity graph using $D = 32$ landmarks. Additionally, its running time and peak memory consumption are the lowest of all approaches, which makes it a suitable candidate for fast and scalable reconstructions. Nonetheless, it is not as robust as STRND regarding performance in different

proximity graphs. For a stochastic decay graph (Tables S10, S13) its local quality metric is slightly worse and for the Delaunay graph (Tables S9, S12) both local and global quality metric are substantially lower.

We further investigate the effect of the number of landmarks on the accuracy scores in order to see if a poor parameter choice led to non-representative results. Although the purpose of landmark nodes is to decrease overall computational complexity, increasing the number of landmarks can have an impact on how well the images can be reconstructed. Indeed, we find that a greater number of landmarks positively affect the accuracy scores (Fig.S8). This effect is clear for Delaunay proximity graphs and in particular for the global CPD quality metric, where an increase in landmarks benefits the accuracy score. However, such scores are still considerably low when compared to other approaches. Conversely, high accuracy scores are shown for the KNN and Stochastic Decay graphs, but the impact of a higher number of landmarks seems more subtle. Table S14 shows the effect of the number of landmarks on empirical computational complexity. Although increasing landmark nodes might have a slight positive effect on accuracy scores, at the same time it can substantially increase computational complexity.

All approaches involving shortest-path computations show a high space complexity as a $N \times N$ shortest-path distance matrix has to be stored and processed by a manifold learning algorithm. The system size quadratic dependence becomes problematic as N grows, leading to memory allocation issues and increasing running times. An exception to this behavior is Landmark Isomap, as it only requires a markedly less computationally demanding $N \times D$ matrix, which can be computed through single-source shortest path algorithms. Possible workarounds to this problem might involve distance matrix pruning and sampling, e.g., establishing a maximum shortest-path threshold or randomly sampling matrix values.

STRND shows the best robustness to different proximity graph types, as it has a similar accuracy and complexity score for different proximity graphs both for 2 and 3 dimensions (Tables S8-S13). This behavior is not as prevalent for other approaches as they show lower robustness concerning accuracy scores and computational complexity.

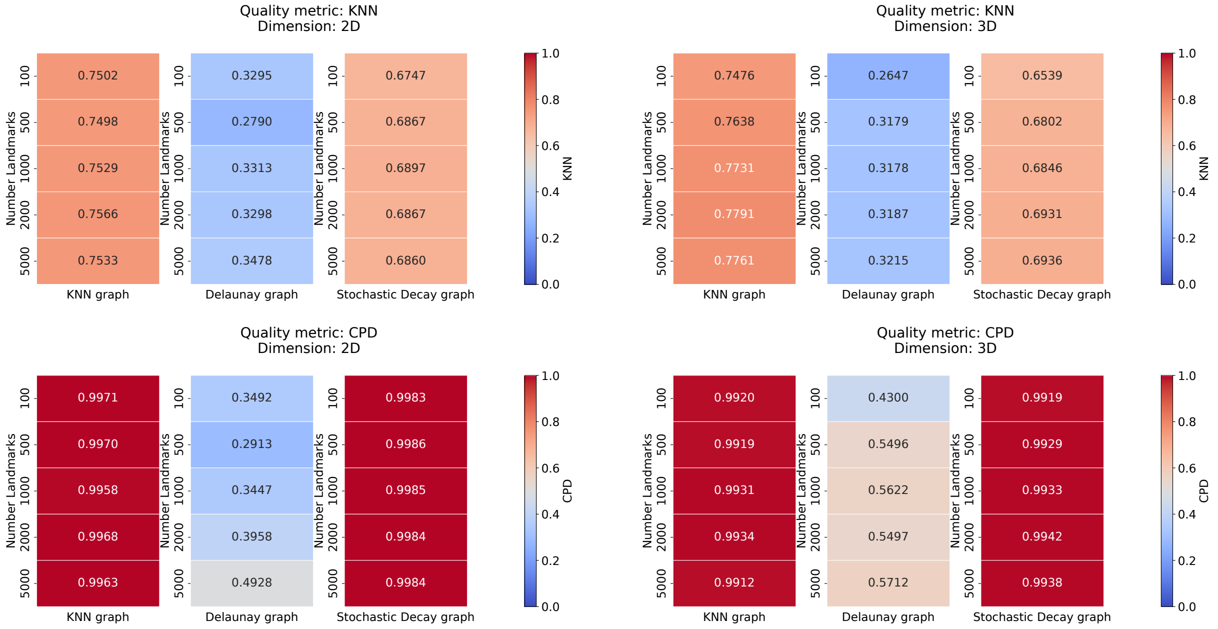

Fig. S8 Effect of the number of landmarks on the accuracy scores for the Landmark Isomap approach regarding the KNN graph, the Delaunay graph and the Stochastic Decay graph for both 2 and 3 dimensions.

Table S14 Landmark Isomap: averaged empirical computational complexity for a different number of landmarks. Each number of landmarks is averaged over 6 samples.

Number of Landmarks	Time (s)	Peak memory (MB)
100	6.3663	33.7942
500	27.5251	72.4396
1000	59.0346	147.6781
2000	156.4137	308.3846
5000	682.9074	1451.1691

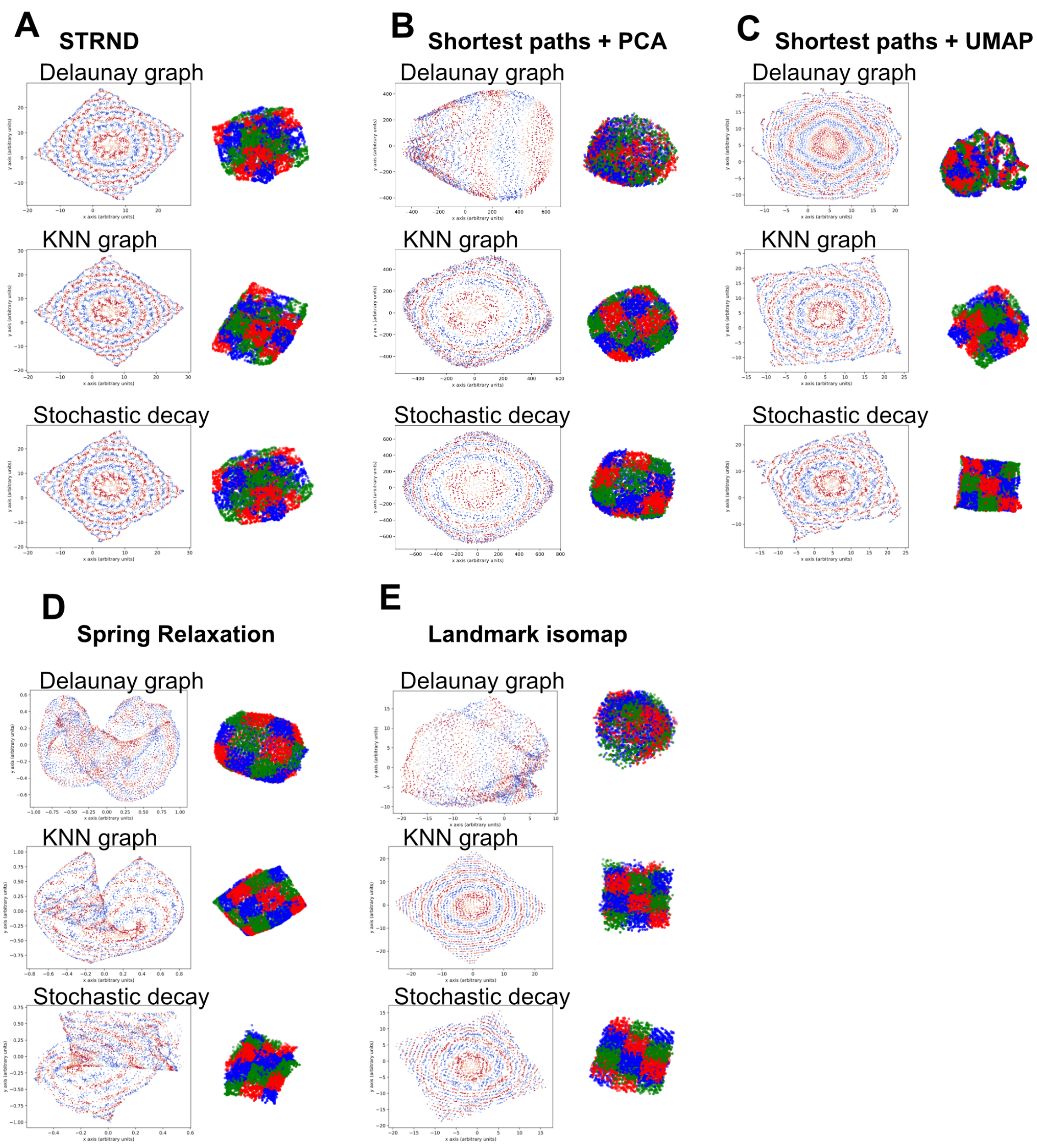

Fig. S9 Visual comparison of reconstruction methods in 2D and 3D for Delaunay, KNN, and stochastic decaying proximity graph patterns for STRND (A), Shortest Paths + PCA (B), Shortest Paths + UMAP (C), Spring Relaxation (D), and Landmark Isomap (E).

4 Code Availability

The full code including STRND and other approaches can be found at `https://github.com/DavidFernandezBonet/ImageRecovery`, including a demo Jupyternotebook that demonstrates use cases and acts as a tutorial for new users. It is also possible to install it as a Python package using *pip install ImageRecovery*.

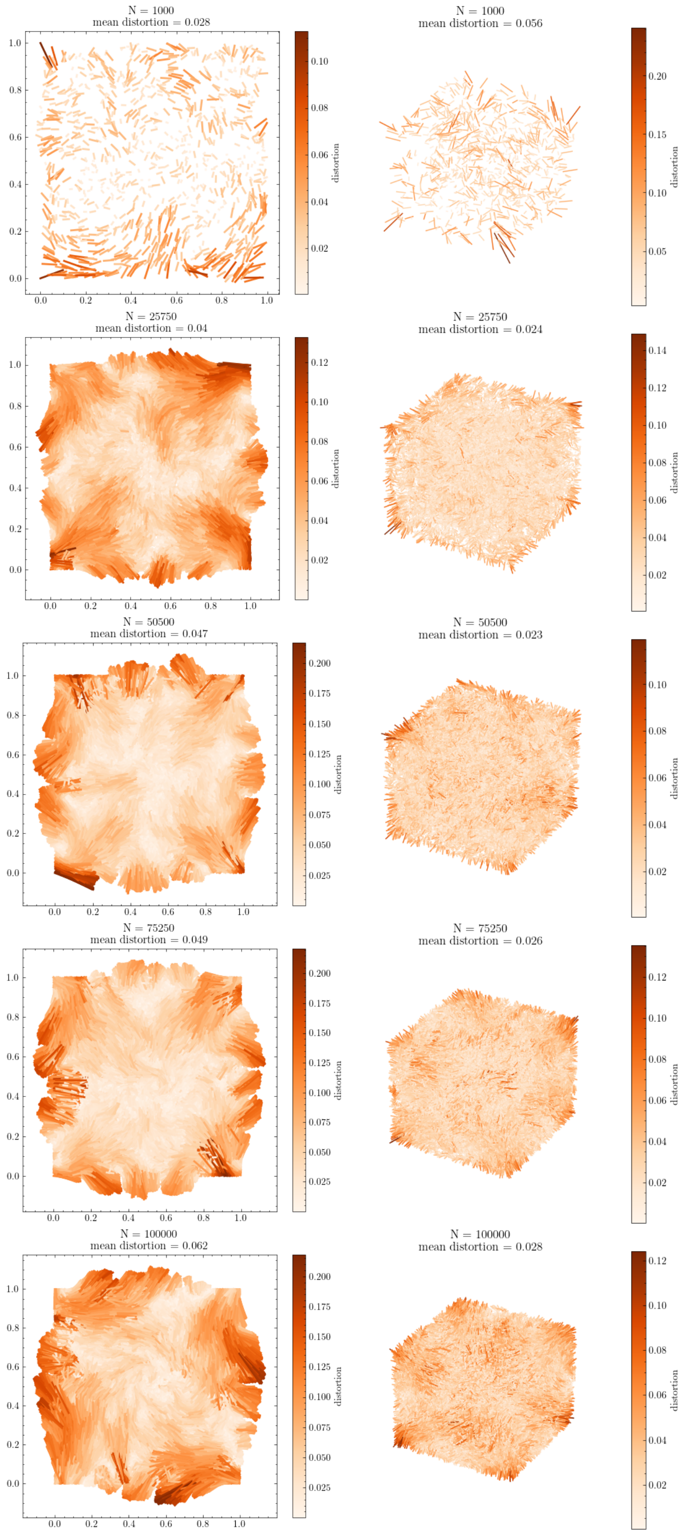

Fig. S10 Distortion plots for every system sized displayed in Fig.3 B for 2 and 3 dimensions.

Notes and references

1 I. T. Hoffecker, Y. Yang, G. Bernardinelli, P. Orponen and B. Högberg, *Proceedings of the National Academy of Sciences*, 2019, **116**, 19282–19287.

2 T. E. Schaus, S. Woo, F. Xuan, X. Chen and P. Yin, *Nature communications*, 2017, **8**, 1–9.

3 J. I. Glaser, B. M. Zamft, G. M. Church and K. P. Kording, *PloS one*, 2015, **10**, e0131593.

4 A. A. Boulgakov, E. Xiong, S. Bhadra, A. D. Ellington and E. M. Marcotte, *bioRxiv*, 2018, 470211.

5 J. A. Weinstein, A. Regev and F. Zhang, *Cell*, 2019, **178**, 229–241.

6 N. Gopalkrishnan, S. Punthambaker, T. E. Schaus, G. M. Church and P. Yin, *bioRxiv*, 2020.

7 L. Greenstreet, A. Afanassiev, Y. Kijima, M. Heitz, S. Ishiguro, S. King, N. Yachie and G. Schiebinger, *bioRxiv*, 2022.

8 M. Balasubramanian and E. L. Schwartz, *Science*, 2002, **295**, 7–7.

9 S. T. Roweis and L. K. Saul, *science*, 2000, **290**, 2323–2326.

10 M. Belkin and P. Niyogi, *Neural computation*, 2003, **15**, 1373–1396.

11 B. Perozzi, R. Al-Rfou and S. Skiena, Proceedings of the 20th ACM SIGKDD international conference on Knowledge discovery and data mining, 2014, pp. 701–710.

12 J. Tang, M. Qu, M. Wang, M. Zhang, J. Yan and Q. Mei, Proceedings of the 24th international conference on world wide web, 2015, pp. 1067–1077.

13 A. Agrawal, A. Ali, S. Boyd *et al.*, *Foundations and Trends® in Machine Learning*, 2021, **14**, 211–378.

14 A. Grover and J. Leskovec, Proceedings of the 22nd ACM SIGKDD international conference on Knowledge discovery and data mining, 2016, pp. 855–864.

15 L. McInnes, J. Healy and J. Melville, *arXiv preprint arXiv:1802.03426*, 2018.

16 W. Gu, A. Tandon, Y.-Y. Ahn and F. Radicchi, *Nature Communications*, 2021, **12**, 3772.

17 A. Myronenko and X. Song, *IEEE transactions on pattern analysis and machine intelligence*, 2010, **32**, 2262–2275.

18 T. M. Fruchterman and E. M. Reingold, *Software: Practice and experience*, 1991, **21**, 1129–1164.

19 A. Hagberg, P. Swart and D. S Chult, *Exploring network structure, dynamics, and function using NetworkX*, Los alamos national lab.(lanl), los alamos, nm (united states) technical report, 2008.

20 M. A. Cox and T. F. Cox, *Handbook of data visualization*, Springer, 2008, pp. 315–347.

21 X. Zhu, X. Li, S. Zhang, Z. Xu, L. Yu and C. Wang, *IEEE Transactions on Multimedia*, 2017, **19**, 2033–2044.
